# Early Biomarkers Associated with P53 Signaling for Acute Radiation Injury

**DOI:** 10.3390/life12010099

**Published:** 2022-01-11

**Authors:** Weihong Li, Shixiang Zhou, Meng Jia, Xiaoxin Li, Lin Li, Qi Wang, Zhenhua Qi, Pingkun Zhou, Yaqiong Li, Zhidong Wang

**Affiliations:** 1 Graduate Collaborative Training Base of Academy of Military Sciences, Hengyang Medical School, University of South China, Hengyang 421001, China; lwh_leity@163.com; 2 Beijing Key Laboratory for Radiobiology, Department of Radiobiology, Beijing Institute of Radiation Medicine, Beijing 100850, China; zhoushixiang104@163.com (S.Z.); jiameng7659@163.com (M.J.); ldx217@yeah.net (X.L.); longyun0515@163.com (L.L.); wqi619@126.com (Q.W.); tjuqzh@163.com (Z.Q.); birm4th@163.com (P.Z.)

**Keywords:** P53, radiation injury biomarker, response to ionizing radiation

## Abstract

Accurate dose assessment within 1 day or even 12 h after exposure through current methods of dose estimation remains a challenge, in response to a large number of casualties caused by nuclear or radiation accidents. P53 signaling pathway plays an important role in DNA damage repair and cell apoptosis induced by ionizing radiation. The changes of radiation-induced P53 related genes in the early stage of ionizing radiation should compensate for the deficiency of lymphocyte decline and γ-H2AX analysis as novel biomarkers of radiation damage. Bioinformatic analysis was performed on previous data to find candidate genes from human peripheral blood irradiated in vitro. The expression levels of candidate genes were detected by RT-PCR. The expressions of screened DDB2, AEN, TRIAP1, and TRAF4 were stable in healthy population, but significantly up-regulated by radiation, with time specificity and dose dependence in 2–24 h after irradiation. They are early indicators for medical treatment in acute radiation injury. Their effective combination could achieve a more accurate dose assessment for large-scale wounded patients within 24 h post exposure. The effective combination of p53-related genes DDB2, AEN, TRIAP1, and TRAF4 is a novel biodosimetry for a large number of people exposed to acute nuclear accidents.

## 1. Introduction

Timely estimation of radiation dose and classification of damage for large numbers of wounded in the early stage of nuclear accidents will effectively guide the application of treatment plans and improve the capacity of treatment and rescue [1,2,3].

The 0.5 Gy irradiation will cause a slight decline of peripheral blood lymphocytes counts and recover spontaneously. A total of 2 Gy irradiation can cause obvious decline of peripheral blood lymphocyte counts and inhibition of hematopoietic function, which usually requires medical treatment. The dose of radiation up to 6 Gy will lead to gastrointestinal symptoms and multiple organs injury (MOI). These patients need multiple organ support therapy from corresponding departments [4,5]. Furthermore, patients irradiated 10 Gy will suffer multi-organ syndrome (MODS) which may result in multi-organ failure (MOF) ultimately, which is irreversible [4,5,6]. They should be transferred to a critical care medicine center. Therefore, it is essential to classify the wounded accurately and rapidly for deciding the treatment of the wounded. Nuclear radiation accidents usually happen accidentally, and the exposed individual will not carry physical dosimeters. Thus, biological dose estimation technology is crucial for the classification and dose assessment of wounded after nuclear accidents. Currently, applied biological dose estimation methods mainly include lymphocyte chromosome aberration analysis, lymphocyte count analysis, lymphocyte γ-H2AX analysis, and so on [7,8,9,10,11]. Among them, lymphocyte chromosome aberration analysis is considered as the “gold standard” for biological dose estimation of radiation for its higher specificity and accuracy. However, high requirements for operators are needed, including more than 48 h cell culture before specimen preparation and analysis. Furthermore, the upper limit of dose detection is 5–6 Gy [12,13]. Ionizing radiation causes a significant decline in peripheral blood lymphocytes, and the degree is dramatically related to the dose. The lymphocyte count analysis within 12 to 48 h after irradiation is used for classification and preliminary dose estimation of patients with radiation injury. However, there are large differences between individuals, resulting in low accuracy [14,15,16]. Since γ-H2AX is recognized as a key biomarker of DSB and has great specificity to ionizing radiation, the γ-H2AX foci analysis is widely used for identifying ionizing radiation. However, the window period of γ-H2AX foci change is short. It appears several minutes after irradiated, the peak comes at 1 h and starts to decrease. Moreover, the operation is complicated and cannot achieve high-throughput detection, which limit its wide application [17,18,19]. In addition, peripheral blood lymphocyte count analysis can be used for triage in large number of patients, and the accurate dose can only be estimated by chromosome aberration analysis. According to the technical characteristics of both methods, it is impossible to achieve patient classification and accurate dose assessment only by lymphocyte count analysis or chromosome aberration analysis in the early post-accident period.

DNA damage and apoptosis are the most basic and critical aspects of the biological effects of radiation and can be used as markers of injury following exposure to ionizing radiation [20,21,22,23,24]. Cells will initiate apoptosis immediately, once DNA damage repair fails [25,26]. Furthermore, 0.5–1 Gy irradiation can induce DNA damage and apoptosis for peripheral blood lymphocytes which are very sensitive to irradiation [27,28]. P53 signaling pathway plays an extremely important role in both DNA damage repair and the initiation of apoptosis [24]. Specifically, DNA double strand breaks (DSBs) induced by radiation activate ataxia telangiectasia mutation (ATM) [29,30], which phosphorylate and activate checkpoint kinase Chk2 [31]. Consequently, p53 is activated and the stability of p53 is enhanced, which result in cell cycle arrest at G1/S phase and provide a critical opportunity for cells to restore genomic integrity prior to DNA replication. Repairable DNA damage tends to be transient, whereas sustained p53 activation will induce the cells to initiate apoptotic program [27,32,33]. P53 signal plays an important regulatory role in the whole process of ionizing radiation-induced DNA damage and apoptosis in most exposed tissues and cells. Enhanced P53 activity is a key marker for DNA damage and cell cycle arrest. Thus, the significant changes of radiation-induced P53 signaling related genes in the early stage of ionizing radiation should be served as novel biomarkers of radiation damage.

In our previous study, a variety of radiation sensitive genes were found in human peripheral blood after 2 h post-irradiation [34]. We then further investigate the early sensitive genes which were mainly related to the P53 signaling pathway. Four genes (DDB2, AEN, TRIAP1, and TRAF4) were identified and stable real-time quantitative fluorescence PCR detection methods were established respectively, to verify their radiation sensitivity and baseline levels in healthy population. Our results showed that the levels of 4 genes in peripheral blood were dose and time dependent significantly at different time and dose range within 24 h after exposure. Further studies indicated that the baseline levels of the four genes in healthy population could not be affected by gender and age. Taken together, we assessed rough thresholds for each gene at different detection time points as guidelines for medical treatment and combined four genes according to their different change patterns in the early radiation stage. We aim to build an effective radiation dose estimation model, which assist to dose estimation of large quantities of people exposed to nuclear radiation.

## 2. Materials and Methods

### 2.1. Human Peripheral Blood Sample Collection

Peripheral blood samples were collected from 32 healthy volunteers aged between 20 to 50 years (female: 15, male: 17). None of them had a history of smoking, drinking, acute or chronic diseases, and no X-ray has been exposed. Blood samples were collected intravenously with anticoagulant EDTA-K2.

### 2.2. Radiation and Culture In Vitro

The 42 mL whole human peripheral blood sample was incubated in a water bath at 37 °C to simulate the in-vivo environment, and divided into 7 groups and irradiated in 0 Gy, 0.5 Gy, 1 Gy, 2 Gy, 4 Gy, 6 Gy, and 10 Gy respectively with ^60^Co-γ radiation at a dose rate of 1.14 Gy/min. The samples were cultured at a constant temperature in an incubator at 37 °C after irradiation, and collected at 0 h, 2 h, 4 h, 8 h, 12 h, and 24 h after irradiation. Total RNA was extracted for subsequent detection.

### 2.3. RNA Extraction and QRT-PCR

Primer pairs and probes for each gene were designed and synthesized by Gemma Company (Shanghai) according to the genetic information retrieved from GenBank. The specific sequence and amplification product size are detailed in (Supplementary Table S3).

Concentration and purity of RNA were extracted from blood samples using RNAprep Pure Blood Kit (QIAGEN, Hilden, Germany), were quantitated by NanoDrop 2000 (Thermo Fisher Scientific, Rockford, IL, USA), and the integrity was detected by 1.5% agarose gel electrophoresis. The PrimeScriptTM RT Reagent Kit with gDNA Eraser was used to reverse transcriptional cDNA as a template.

According to the genetic information retrieved from GenBank, the primers and probes of each gene were designed and synthesized by Gemma Company, Shanghai. ITaq^TM^ Universal SYBR Green Supermix (BIO-RAD) was used for QRT-PCR analysis through TaqMan probe two-step Real Time PCR. The relative levels of candidate gene expression were calculated using the formula 2^−ΔΔCT^ as described in the user manual.

### 2.4. Plasmid Construction and Standard Curve Drawing

Total RNA was extracted from the collected peripheral blood samples and reverse-transcribed into cDNA using PRIME Scripttm RT Reagent Kit with GDNA Eraser Kit. The target gene fragments of TRAF4, DDB2, AEN, TRIAP1, and β-actin were amplified using reverse transcribed cDNA as the template, and then linked to pMD-18T vector and confirmed by sequencing. The plasmid concentration measured by spectrophotometer was converted into molar concentration. The genes β-actin, TRAF4, DDB2, POLH, GADD45A, and TNFSF9 were amplified by MYIQTM2 real-time quantitative PCR using plasmid standards of different concentrations as templates. The mole number of the standard plasmid was transformed into the copy number of the plasmid according to the formula Copies/μL = (6.02 × 10^23^) × (ng/μL × 10^−9^)/(DNA Length × 660) and then logarithm transformed as the abscissa of the curve. The ordinate of the curve is the CT values at different concentrations measured experimentally. The standard curves of β-actin, TRAF4, DDB2, GADD45A, and TNFSF9 genes were plotted according to the above methods. The equation of the standard curve is y = −ax + b.

### 2.5. Statistical Analysis

The results of the experiment used the relative quantitative analysis method of 2^−∆∆CT^. β-actin was used as the reference gene to measure the mRNA expression level of radiation-sensitive genes. PCR data analysis and comparison between groups were performed by SPSS18.0 (IBM). Bilateral One-way analysis of variance test was used, with *p* < 0.05 as significant difference. The dose-time response relationships of individual radiation-responsive genes were analyzed by linear or nonlinear regression methods. PLS regression analysis was used to establish a dosimetry model of gene expression. R2 values were used to determine the goodness of fit for all models and assess the combination of genes.

## 3. Results

### 3.1. Ionizing Radiation Induced the Expression of P53 Signaling Related Genes including DDB2, AEN, TRIAP1 and TRAF4

**Figure 1:**
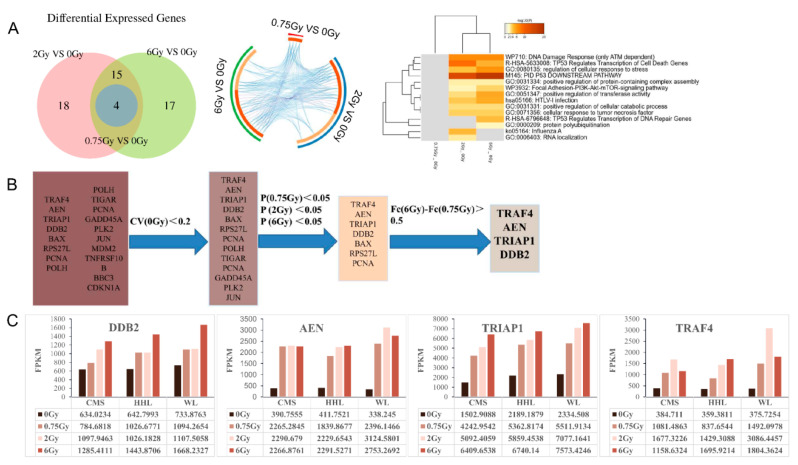
Analysis of differentially expressed genes in human peripheral blood 2 h after γ-ray irradiation. (**A**) Overlap analysis was performed on differentially expressed genes in 2 h after different doses of irradiation. Functional annotation analysis were performed on these differentially expressed genes. On the outside, each arc represents the identity of each gene list, each deep orange arc inside the diagram represents a gene that appears in multiple lists, and the light orange arc represents a single gene in that list. The purple line connects differentially expressed genes that were common to the different dose groups and the blue line connects differentially expressed genes enriched in the same GO term. All statistically enriched were hierarchically clustered into a tree and 0.3 kappa score was applied as the threshold to cast the tree into term clusters; the heatmap cells are colored by their *p*-values, white cells indicate the lack of enrichment for that term in the corresponding gene list. (**B**) Screening procedures for candidate biomarkers. Among the 17 genes associated with P53, the genes whose coefficient of variation (CV) > 2 and Fold Change (FC) in 0.75 Gy, 2 Gy and 6 Gy is not significant compared to 0 Gy were eliminated. Finally, we selected four genes whose ratio of maximum to minimum of Fold Change in 0.75 Gy, 2 Gy, and 6 Gy was greater than 0.5. (**C**) Expression levels of P53 related candidate genes in human peripheral blood before and after different doses of irradiation.

We conducted an in-depth analysis of NimbleGen genome-wide chip data from the peripheral blood samples of 3 healthy adults with doses of 0 Gy, 0.75 Gy, 2 Gy, and 6 Gy at 2 h after irradiation. For each dose group, we screened genes with expression fold change greater than 2 times in the three samples as differentially expressed genes (Figure 1A and Supplementary Table S1). Four genes, including AEN, BBC3, TRAF4, and TRIAP1, were significantly increased in all three samples after 0.75 Gy radiation. Meanwhile, all of them were shown significant difference in 2 Gy and 6 Gy dose groups. The numbers of differentially expressed genes were 37 and 36 in 2 Gy and 6 Gy groups, in which 19 genes were same. Subsequently, functional annotation analysis was conducted for the differential expressed genes of each dose group. The results showed that the differentially expressed genes in 0.75 Gy and other dose groups were enriched into few signal pathways. The most significant pathway enriched was P53 downstream pathway, as well as TP53 regulates transcription of cell death genes and TP53 regulates transcription of DNA repair genes. These results indicated that P53-related signaling pathways significantly changed in peripheral blood after irradiation (Figure 1A).

We then compared all differentially expressed genes enriched in TP53-related signaling pathways in different databases and our results revealed a total of 17 differentially expressed genes were involved in TP53-related signaling pathways (Supplementary Table S2). We then explored the levels of the 17 differentially expressed genes, and four genes with variation coefficients greater than 0.2 in normal samples were excluded. The expressions of 7 genes were significantly up-regulated in all radiated groups. Finally, only 4 target genes with dose dependence were screened. The detailed process of filtering and expression levels of target genes is shown in the Figure 1B,C. Therefore, our study stepped to the four differentially expressed genes: DDB2, AEN, TRIAP1, and TRAF4, and explored their feasibility as early biomarkers response to ionizing radiation damage.

### 3.2. Time and Dose Effect Analysis of Ionizing Radiation Induced Gene Expressions

First, we established real time PCR detection method for DDB2, AEN, TRIAP1, and TRAF4 genes. We used peripheral blood cDNA as a template to optimize the amplification temperature conditions for primers of DDB2, AEN, TRIAP1, TRAF4, and internal reference gene β-actin (Supplementary Figure S1A). Therefore, we chose 60 °C as the optimal annealing temperature for all 5 genes. Then, standard plasmid were constructed respectively and diluted by a 10-fold gradient for real-time quantitative PCR. As shown in Supplementary Figure S1B, we obtained uniform amplification curves with a CT interval of about 3.33, and all had strong fluorescence for all above 5 plasmids. Subsequently, the standard curve is shown in Supplementary Figure S1C. The results showed that there was an obvious linear relationship between the plasmid copy numbers and CT values.

**Figure 2:**
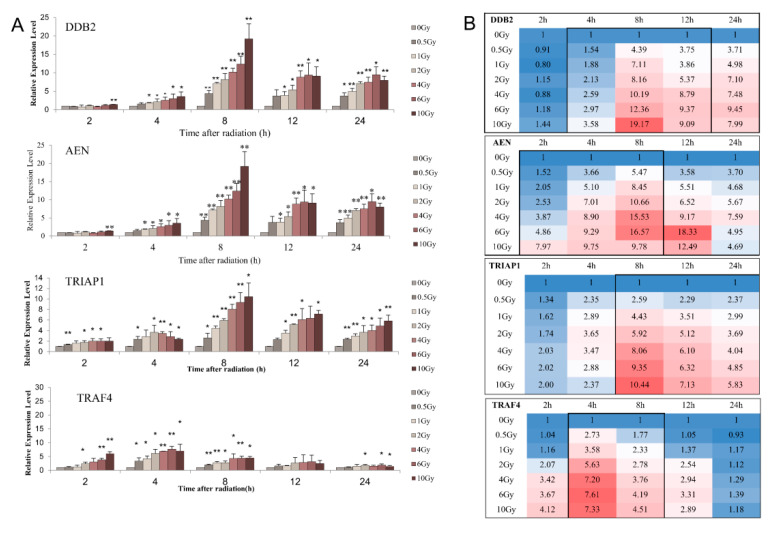
Analysis on time and dose effects of radiation sensitive genes. (**A**) The histogram shows the time effect and dose effect of DDB2, AEN, TRIAP1, and TRAF4 after irradiation; * *p* < 0.05, ** *p* < 0.01. (**B**) The heat map shows the response time window and change pattern of DDB2, AEN, TRIAP1, and TRAF4 after exposure to ionizing radiation. Cells are colored by their fold changes, black windows frame the time range of the genes response to radiation dose.

In order to analyze the time and dose effects of the four candidate genes after irradiation, human venous blood was irradiated with 0–10 Gy γ-rays in vitro, and samples were collected at 0 h, 2 h, 4 h, 8 h, 12 h, and 24 h after irradiation. The expression of DDB2 gene showed a significant dose-dependently increase at four time points from 4 h to 24 h after irradiation, and peaked at 8 h after irradiation. At 24 h after irradiation, the expression of DDB2 was still significantly higher in the 0.5 Gy irradiation groups than unirradiated group (Figure 2A). The expression of AEN gene showed a significant increase dose-dependently at all four time points from 2 to 12 h after exposure, and began to increase after 2 h exposure, peaked at 8 h to 12 h after exposure, and then fell from 24 h exposure. However, it was still higher than that of the unexposed group (Figure 2A). The expression of TRIAP1 gene was significantly up-regulated from 2–24 h after irradiation, and peaked at 8 h after irradiation. At 2 h and 4 h after irradiation, the expression level of TRIAP1 increased significantly in different dose groups, but there was no significant dose-dependent relationship. At 8 h, 12, and 24 h after irradiation, the expression level of TRIAP1 at each dose point was significantly higher than that in the unirradiated group, showing a dose-dependent increase (Figure 2A, middle, and bottom). The increase of TRAF4 gene expression mainly occurred at 2–8 h after irradiation, and there was no significant change at 12 and 24 h after irradiation. At 2 h after radiation, the expression level of TRAF4 was mainly upregulated in the 6 Gy and 10 Gy groups, while the expression level of TRAF4 gene showed an increasing trend after 4 Gy and below irradiation, but there was no statistical significance. At 4 h after exposure, the expression level of TRAF4 reached a peak and increased dose-dependently, then began to fall back at 8 h after exposed and fell back to the normal level at 24 h after exposure (Figure 2A bottom).

Comprehensive analysis showed that those four genes had characteristic patterns in the time range and dose range in the early stage response to radiation. As shown in Figure 2B, DDB2 had significant time and dose effects after 4 to 8 h post irradiation. AEN showed a significant dose effect from 2 to 8 h post-exposure. The TRIAP1 pair dose was significant from 8 h to 24 h post-exposure. The detection window period for TRAF4 was shorter, at 4 to 8 h after irradiation.

### 3.3. Expressions of DDB2, AEN, TRIAP1, and TRAF4 in Healthy Population

**Figure 3:**
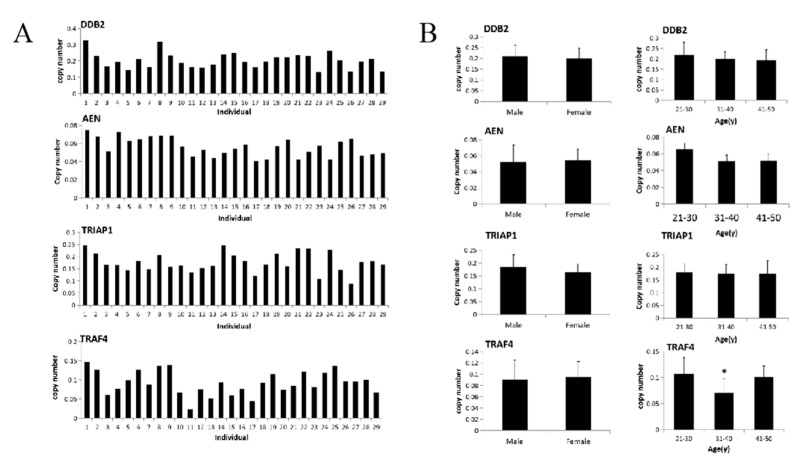
Baseline level analysis of DDB2, AEN, TRIAP1, and TRAF4 in healthy population (**A**) Collected the venous blood from 29 healthy volunteers (age ranging from 20 to 50 years old, male: 15 and female: 14), and analyzed the expression levels of DDB2, AEN, TRIAP1, and TRAF4. (**B**) The effects of gender and age on DDB2, AEN, TRIAP1, and TRAF4 gene expression were analyzed (* *p* < 0.05).

To further evaluate the feasibility of DDB2, AEN, TRIAP1, and TRAF4 as biomarkers of radiation injury, we collected venous blood from 29 healthy volunteers (age ranging from 20 to 50 years old, male: 15 and female: 14) to conduct a preliminary analysis of the expression levels of these four genes in healthy population. The results showed that the distribution of baseline levels of four genes in healthy population was uniform and stable, without large fluctuations (Figure 3A). The average copy number of DDB2, AEN, TRIAP1, and TRAF4 genes in 29 healthy people were DDB2: 0.204 ± 0.049, AEN: 0.056 ± 0.010, TRIAP1: 0.170 ± 0.031, TRAF4: 0.0924 ± 0.031 respectively; and the fluctuation ranges were DDB2: 0.103~0.326, AEN: 0.041–0.075, TRIAP1: 0.101–0.248, TRAF4: 0.023~0.146 respectively. Further analysis showed that there was no significant difference of these four genes between male and female. The levels in male and female groups were DDB2: 0.208 ± 0.053, 0.200 ± 0.047, *p* = 0.695; AEN: 0.055 ± 0.011, 0.057 ± 0.010, *p* = 0.278; TRIAP1: 0.174 ± 0.033, 0.166 ± 0.029; *p* = 0.445; TRAF4: 0.091 ± 0.035, 0.094 ± 0.028, *p* = 0.787 (Figure 3B left). Finally, 29 samples were divided into 3 groups according to age. We aim to investigate whether the genes expressions were affected by age, including 10 people aged 21 to 30 years, 10 people aged 31 to 40 years and 9 people aged 41 to 50 years. The expression levels of DDB2 in all age groups were 0.219 ± 0.062, 0.199 ± 0.034 and 0.194 ± 0.049 (*p* > 0.05), and there was no significant difference among all groups. The expression levels of AEN in different age groups were 0.066 ± 0.007, 0.051 ± 0.008 and 0.052 ± 0.008. There was no significant difference in pairwise comparison among all groups (*p* > 0.05). The expression levels of TRIAP1 gene in all age groups were 0.179 ± 0.033, 0.170 ± 0.031 and 0.160 ± 0.029 (*p* > 0.05, Figure 3B right). The expression levels of TRAF4 gene in 3 groups were 0.107 ± 0.032, 0.071 ± 0.027 and 0.100 ± 0.022. The *p* values comparison between the 31–40 years group and the other two groups were 0.017 and 0.021 (Figure 3B right).

### 3.4. DDB2, AEN, TRIAP1, and TRAF4 Can Be Used as Early Indicators for Medical Treatment in Acute Radiation Injury

**Figure 4:**
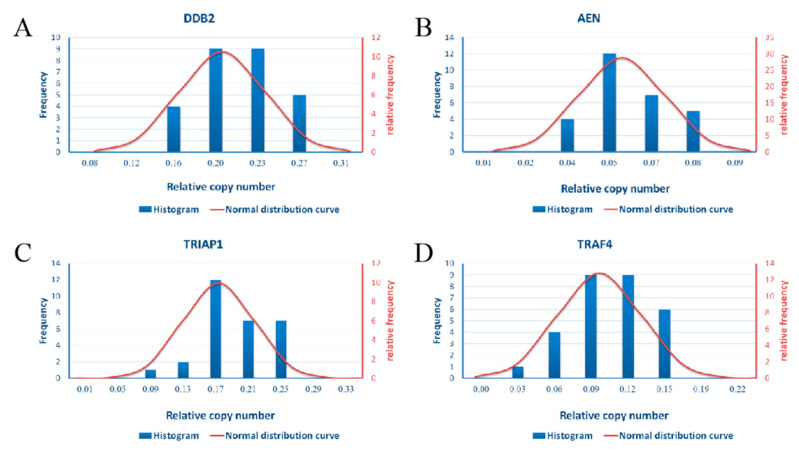
The normal distribution curve of 4 genes’ expression level in population was drawn. (**A**) The total normal distribution curves of DDB2 expressed in population. (**B**) The total normal distribution curves of AEN expressed in population. (**C**) The total normal distribution curves of TRIAP1 expressed in population. (**D**) The total normal distribution curves of TRAF4 expressed in population.

For the stability of the expression levels of four genes in healthy population, we plotted the population normal distribution curve (Figure 4) and estimated the 95% confidence interval based on central limit theorem. The results are shown in Table 1. The central values, standard deviations, and 95% confidence intervals of four genes in healthy population are DDB2:Mean = 0.196, STD = 0.038, CI = 0.120~0.271; AEN:Mean = 0.051, STD = 0.014, CI = 0.023–0.079; TRIAP1:Mean = 0.176, STD = 0.040, CI = 0.096~0.256; TRAF4:Mean = 0.092, STD = 0.031, CI = 0.030~0.154. The coefficient of variation and the fold change of the maximum and minimum values within the confidence interval were respectively DDB2:CV = 0.194, FC_bg_ = 2.264; AEN:CV = 0.275, FC_bg_ = 3.435; TRIAP1:CV = 0.228, FC_bg_ = 2.677; TRAF4:CV = 0.336, FC_bg_ = 5.106. Once the fold changes of the above genes before and after exposure beyond the multiple of FC_bg_ or the copy number of expressed exceeds the 95% confidence interval in human peripheral blood changes that may indicate that the individual maybe exposed to ionizing radiation.

Exposure to moderate doses (2–6 Gy) of radiation leads to a hematopoietic sub-syndrome (H-ARS) [4,6], which usually required for medical treatment, we then further compared the FC_bg_ of DDB2, AEN, TRIAP1, and TRAF4 expression and the ratio of maximum to minimum value within the confidence interval of baseline level at different time points after irradiation in healthy population. In combination with the data in Figure 3B, we aim to find early indicators for medical treatment in exposed patients. The minimum of fold changes was chosen among groups radiated more than 2 Gy in each time point compared with the FC (bg), and the larger one was selected as FC value. We can identify the individual who have been exposed in radiation at more than 2 Gy when the upregulation of gene expression exceeds the corresponding FC, and then medical treatment is needed. Correspondingly, we took the product of FC and the central value of normal population (μ) as the approximate threshold for considering medical treatment. In other words, once the number of copies of the target gene is more than Copies = FC × μ, it means the indivual may be diagnosed with acute radiation syndrome (ARS), and symptomatic treatment was needed. Their respective threshold values could be recognized at 2–24 h after irradiation (Table 2). In the case of sufficient medical resources, if the expression level of more than one of the four genes reaches the threshold, attention should be paid and immediate medical treatment should be given. Medical treatment should be considered if there are more than two time points within 2–24 h after exposure, at which the dosage reach the indications for medical treatment.

Based on existing experience, much higher doses of radiation will lead to discrete sub-syndromes in the gastrointestinal tract (6–10 Gy; destruction of the intestinal tissue, dehydration, electrolytes imbalance) and multiple organs injury (MOI). These patients need hematologists, dermatologists, neurologists, gastroenterologists, etc. to give specific recommendations regarding the organ system of their competence [4,5]. Thus, we need to screen patients with MOI as early as possible. The minimum of the fold change of expression in groups radiated more than 6 Gy which was significantly higher than that in the groups less than 4 Gy were selected as the value of FC at each detection time point. The product of FC and μ, the central value of population normal distribution curve was used as the approximate threshold to warning that the patient was likely to have exposed to radiation more than 6 Gy and it will cause MOI. Thus, these patients should be sent to a major hospital with all the appropriate specialties available 7 days a week, 24 h a day. Concrete values are shown in Table 3.

Patients irradiated more than 10 Gy, will suffer incurable neurovascular system injury and multi-organ Syndrome (MODS)-ultimately resulted in multi-organ Failure (MOF), which is irreversible [4,5,6]. The minimum fold change of gene expression in 10 Gy, but significantly higher than that in the 4 Gy and below groups were selected as the value of FC. The product of FC and μ, the central value of population normal distribution curve was used as the approximate threshold to warning that the patient was likely to have exposed to radiation more than 10 Gy and will caused MOF. Maybe they should be transferred to a large hospital specializing in critical care and treatment. Concrete values are shown in Table 4.

### 3.5. Combination of DDB2, AEN, TRIAP1, and TRAF4 Can Be Used for Quantifying Acute Dose

**Figure 5:**
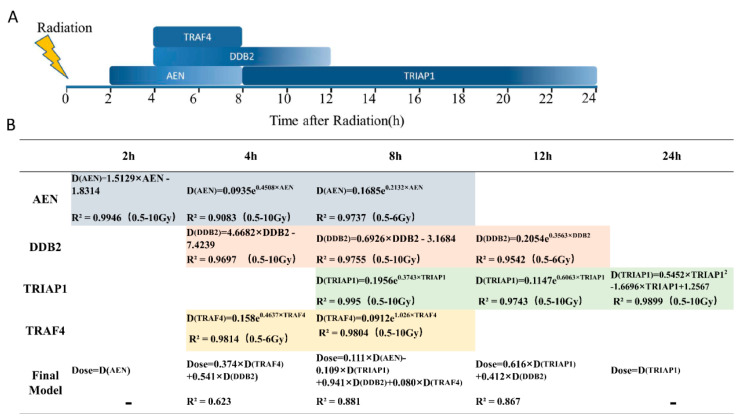
Multiple genes were combined to form a final model to assess the doses of ionizing radiation (**A**) Model of DDB2, AEN, TRIAP1 and TRAF4 response time to ionizing radiation. (**B**) The dose estimation equation was fitted for each gene within the dose range with significant dose dependence at its respective time point, which was taken as a new independent variable to fit the final dose estimation equation by PLS algorithm.

Since DDB2, AEN, TRIAP1, and TRAF4 had significant differences in time window of response to ionizing radiation, the combination of them could perfectly cover all the time periods within a day after exposure (Figure 5A). Therefore, the combination of them at different time periods after radiation were explored for the prediction according to the unique variation patterns. We aim to build a dose assessment model with good accuracy for the exposed dose above 0.5 Gy at any time period within 24 h after radiation. Firstly, the dose estimation equation for each gene was fitted at different time points in time window. The results are shown in Figure 5B: Equations for AEN were Dose2h = 1.5129 × AEN − 1.8314, R^2^ = 0.9946; Dose4h = 0.0935e^0.4508 × AEN^, R^2^ = 0.9083; Dose8h = 0.1685e^0.2132 × AEN^, R^2^ = 0.9737. Equations for DDB2 were Dose4h = 4.6682 × DDB2 − 7.4239, R^2^ = 0.9697; Dose8h = 0.6926 × DDB2 − 3.1684, R^2^ = 0.9755; Dose12h = 0.2054e0.3563 × DDB2, R^2^ = 0.9542. Equations for TRIAP1 were: Dose8h = 0.1956e^0.3743 × TRIAP1^, R^2^ = 0.995; Dose12h = 0.1147e^0.6063 × TRIAP1^, R^2^ = 0.9743; Dose24h = 0.5452 × TRIAP12 − 1.6696 × TRIAP1 + 1.2567, R^2^ = 0.9899. Equations for TRAF4 were: Dose4h = 0.158e^0.4637 × TRAF4^, R^2^ = 0.9814; Dose = 0.0912e^1.026 × TRAF4^, R^2^ = 0.9804. Then we took the fitting formula of each gene as the new independent variables D(DDB2), D(AEN), D(TRIAP1), and D(TRAF4) at 4–12 h after irradiation to fit the final dose estimation equations through PLS fitting algorithm. The results are shown in Figure 5B: Dose4h = 0.374 × D(TRAF4) + 0.541 × D(DDB2), R^2^ = 0; Dose8h = 0.111 × D(AEN) − 0.109 × D(TRIAP1) + 0.941 × D(DDB2) + 0.080 × D(TRAF4), R^2^ = 0.881; Dose12h = 0.616 × D(TRIAP1) + 0.412×D(DDB2), R^2^ = 0.867.

Finally, we compare the model with the existing radiation biodosimetry. The results show that our model can obtain the accurate dose at 5 h after exposure, which is significantly earlier than others. Thus, it would play an important role in medical rescue after nuclear accident.

## 4. Discussion

Current methods of dose estimation are still lacking of speedy and accuracy, in the face of a large number of casualties caused by nuclear or radiation accidents. Therefore, it is urgent to discover new radiation biodosimeter and radiation dose assessment model. They will guide medical personnel to classify injuries and develop effective treatment plans quickly in accidents to minimize casualties.

In our previous study, a variety of radiation sensitive genes were found 2 h after irradiation from human peripheral blood irradiated in vitro [34]. The present study found four sensitive genes DDB2, AEN, TRIAP1, and TRAF4 from the gene chip data. Further biological analysis indicated that they are closely related to P53 signal and could be used as biomarkers of acute irradiation. It has been reported that DDB2 (Damage-specific DNA-binding protein 2) encodes DNA damage-binding protein 2, which is recognized as a radiation sensitive gene [35,36,37]. DDB2 is increased and activated after the body exposing to ionizing radiation, then binds to DNA damage sites and induces the up-regulation of p53 expression level, which eventually participates in the nucleotide excision repair pathway (NER) [38,39,40]. As a radiation sensitive gene, AEN is a direct target gene of p53 regulatory network pathway and could promote apoptosis. AEN is induced by p53 with various DNA damage, and its expression is regulated by the phosphorylation status of all three p53 family members (p53, p63, and p73) [41]. TRIAP1, also known as P53CSV, is an apoptosis suppressor gene, which may bind P53 gene coding sequence [42,43,44]. It is a target gene directly regulated by P53 during DNA damage, cell apoptosis and cell cycle inhibition. In certain stress, p53 regulates TRIAP1 transcription and DNA damage induces endogenous levels of TRIAP1 [45]. TRIAP1 prevents the mitochondrial pathway of apoptosis, and loss of TRIAP1 affects the accumulation of cardiolipin, which is prone to apoptosis, under internal or external stimuli [44]. TRAF4, a member of the TNF receptor-related factor family, encodes a 54 kD connexin consisted of 470 amino acid, is highly expressed in a variety of cancer cells [46,47,48]. TRAF4 has been widely recognized as a common target of the P53 family in previous studies [49,50] due to the presence of binding site approximately 1 KB upstream of the promoter [51]. It has been reported that Mir-29 promotes apoptosis in a p53-dependent manner through TRAF4/AKT/MDM2 pathway in glioma [52]. It is also revealed that TRAF4 interacts with the deubiquitinating enzymes USP10, blocks the binding of p53 to USP10 and induce p53 instability [53,54]. In summary, DNA damage-induced gene DDB2 is significantly up-regulated in the early stage of ionizing radiation, which induces p53 signal activation, and drives the biological processes, such as p53-mediated cell cycle arrest, DNA damage repair and cell apoptosis. As the most significant and direct biological effect of ionizing radiation, the four radiation sensitive genes are closely related to this process.

Rapid reduction of lymphocyte count is one of the main clinical manifestations of acute external irradiation injury. The number of peripheral blood lymphocytes decreased is significantly 12 h after irradiation when irradiation dose is exceeded 0.5 Gy. The degree of reduction was positively correlated with the dose in the range of 0.5~10 Gy [55,56]. At present, exposure dose is mainly estimated based on the absolute value of lymphocytes 12 h or 24–48 h after irradiation, and the applicable is limited to 10 Gy. In our study, the expression levels of DDB2, AEN, TRIAP1, and TRAF4 were significantly increased at 4–12 h, 2–4 h, 8–24 h, and 4–8 h after irradiation in human peripheral blood irradiated at 0.5–10 Gy in vitro, indicating significant dose and time dependence. The R2 of fitting curves were all higher than 0.9. Compared with the traditional lymphocyte counting, our method brings the sampling time forward to 2 h after irradiation. This is of great significance in large-scale emergency rescue. The range of dose detection in our study was 0.5–10 Gy, which is similar to the lymphocyte counting method. However, we could completely predict a higher dose according to the obtained curvilinear equation, within effective time range for detection. Furthermore, all the expression levels of four genes were stable in healthy population, and the difference was found between male and female. Apart from TRAF4, whose expression was slightly lower in the 30–40 age group, the other three genes could not be affected by age. Therefore, the accuracy of our model has more advantage than the traditional lymphocyte count analysis. Moreover, we compared our study with γ-H2AX analysis and lymphocytic chromosome error analysis in Table 5. γ-H2AX has been widely recognized by in vitro and in vivo methodological studies and clinical studies. It has advantages of high sensitivity, as well as disadvantages like short detection window of 1–2 h post-radiation and the upper dose is 5–6 Gy [57,58]. Chromosomal aberration analysis is considered as the gold standard for biological dose estimation due to its high accuracy and specificity [56]. However, the rate of chromosome aberration was basically saturated at 5 Gy, and lymphocytes need to be cultured for 48 to 72 h before analysis. Our dose estimation model covers a wider range of irradiated dose and get evaluated dose as early as 5 h after exposure, so it has greater application value in large-scale nuclear accidents.

With the rapid development of various omics techniques, the study of molecular markers specific for radiation injury has been widely carried out. Potential molecular markers reported by existing studies include small metabolite molecules, coding and non-coding RNA, cytokines, chemokines and other proteins [6,59,60]. Citrulline is the only reliable tissue injury specific biomarker identified by metabonomics in radiobiology [4]. Citrulline is a nitrogen end-product of enteric glutamine metabolism in the small intestine and has been identified as a potential circulating biomarker of radiation-induced gastrointestinal injury and epithelial cell loss. The correlation between radiation-induced epithelial cell loss and plasma citrulline level has been well validated in mice [61,62], and several investigators are working with other animal models to validate this biomarker for radiation injury. In NHPs exposed to lower doses of radiation (5.8 and 6.5 Gy), we did not observe reduction in citrulline levels [63]. In this case, enterocyte damage may not have been substantial enough to significantly lower citrulline. However, exposure to 7.2 Gy of radiation reduced circulating citrulline levels in NHPs [6]. Here have also been many serious attempts to estimate radiation dose exposure using hematological, biochemical, and cytogenetic parameters. Several proteins such as CRP, amylase, cytokines, and growth factors have been investigated for their possible contributions. However, these biological agents have large inter-individual variations and fluctuate as a result of common variables such as inflammation and infection [64]. The expression levels of Mir-200b and Mir-762 in serum of mice exposed to high dose radiation were significantly increased. Some studies have also shown that Mir-30b and Mir-30C in mice are up-regulated after 7 and 10 Gy whole-body γ-irradiation (60 Co, 0.6 Gy/min). Consistent with a single acute exposure, mouse serum Mir-150 was reduced by 50% (total dose 4 Gy) 24 h after fractional radiation exposure [65]. Some lncRNA responded to radiation in a time and dose dependent manner [66,67]. In our study, the combination of four genes response to radiation as early as 2 h after expose and the range of dose detection cover from 0.5 Gy to 10 Gy. Furthermore, apart from TRAF4, whose expression was slightly lower in the 30–40 age group, the baseline levels of the four genes in healthy population could not be affected by gender and age. Therefore, the practicability of our model has more advantage than most of the existing radiation-specific biomarker molecules.

P53-related genes DDB2, AEN, TRIAP1, and TRAF4 with significant radiosensitivity were screened from human peripheral blood irradiated, and this is the first time to investigate the potential biomarkers of ionizing radiation by systematic study. The expression levels of P53 signal-related genes such as DDB2, AEN, TRIAP1, and TRAF4 were significantly upregulated by radiation, with time specificity and dose dependence in 2 h–24 h after irradiation. In addition, the expression levels of DDB2, AEN, TRIAP1, and TRAF4 in healthy population were stable and uniform, with no gender difference and less influenced by age, which could be used as indicators to identify a person who has been exposed to radiation and need medical treatment. The effective combination of the four genes could achieve a more accurate dose assessment for large-scale wounded patients within 24 h post exposure, providing a basis for subsequent treatment plan formulation.

## 5. Conclusions

Our study provides a new model for assisting dose estimation of a large number of exposed patients within 24 h after acute nuclear accident, which compensates the deficiency of lymphocyte decline and γ-H2AX analysis used to assess the dose in the early stage of accidents. With the development of radiation medicine, the range of time and dose detection of the model will be improved continuously, through continuous supplement or modification of novel radiation-sensitive genes according to the characteristics of different genes. Thus, the model is more valuable in medical rescue after nuclear accidents compared with the existing radiation bio-dosimeters.

## Figures and Tables

**Table 1:** Normal distribution parameters of target genes in healthy population. μ: mean; σ: standard deviation; CV: Coefficient of Variation; CI: confidence interval; FC(bg): The maximum of fold change in background.

Item Gene	μ	σ	CV	95% CI	FC(bg)
DDB2	0.196	0.038	0.194	0.120~0.271	2.264
AEN	0.051	0.014	0.275	0.023~0.079	3.435
TRIAP1	0.176	0.040	0.228	0.096~0.256	2.677
TRAF4	0.092	0.031	0.336	0.030~0.154	5.106

**Table 2:** Warning threshold for acute radiation exposure.

Item Gene	2 h		4 h		8 h		12 h		24 h	
	FC	Copies	FC	Copies	FC	Copies	FC	Copies	FC	Copies
DDB2	-	-	2.26	0.44	8.16	1.6	5.37	1.05	7.1	1.39
AEN	3.44	0.18	7.01	0.36	10.7	0.54	6.52	0.33	-	-
TRIAP1	-	-	3.65	0.64	5.92	1.04	5.12	0.9	3.69	0.84
TRAF4	-	-	5.63	0.52	-	-	-	-	-	-

FC: fold change; Copies: the number of copies of the target gene per 100 copies of β-actin.

**Table 3:** Warning threshold for MOI induce by irradiation. FC: fold change; Copies: the number of copies of the target gene per 100 copies of β-actin.

Item Gene	2 h		4 h		8 h		12 h		24 h	
	FC	Copies	FC	Copies	FC	Copies	FC	Copies	FC	Copies
DDB2	-	-	2.97	0.58	12.4	2.42	9.09	1.78	7.99	1.56
AEN	4.86	0.25	9.29	0.47	-	-	12.5	0.64	-	-
TRIAP1	-	-	-	-	9.35	1.64	6.32	1.11	4.85	0.85
TRAF4	-	-	7.33	0.68	-	-	-	-	-	-

**Table 4:** Warning threshold for MOF induce by irradiation. FC: fold change; Copies: the number of copies of the target gene per 100 copies of β-actin.

Item Gene	2 h		4 h		8 h		12 h		24 h	
	FC	Copies	FC	Copies	FC	Copies	FC	Copies	FC	Copies
DDB2	-	-	3.58	0.70	19.17	3.75	-	-	-	-
AEN	7.97	0.41	9.75	0.50	-	-	-	-	-	-
TRIAP1	-	-	-	-	10.44	1.84	7.13	1.25	5.83	1.33
TRAF4	-	-	-	-	5.41	0.50	-	-	-	-

**Table 5:** Time to estimate irradiation dose. TPR: time post-irradiation.

	Dose Range	Sample Collection (TPR)	Sample Processing	Detection and Analysis	Total Time Spent	Time to Get Evaluated Dose (TPR)
Lymphocyte count analysis	0.5 Gy~10 Gy	12 h-25 d	Sample collection 2–3 min	2 min	5 min	12–25 d
γ-H2AX analysis	0.05 Gy~5–6 Gy	1–2 h	Irrigation, incubation and fixation (2 h) Block and incubation (2 h)	Automatically	5–6 h	7–8 h
Lymphocyte chromosome aberration analysis	0.1 Gy~5–6 Gy	48 h-Several decades	Cell culture (48 h) Incubation and fixation (3 h)	Automatically	2–3 days	3–4 days
Combination of P53-related genes	0.5 Gy~10 Gy	2–24 h	RNA extraction and reverse transcription(1.5 h)	RT-PCR 1.5 h	3 h	5 h–27 h

## Data Availability

The datasets used or analysed during the current study are available from the corresponding author on reasonable request.

## Supplementary Materials

The following supporting information can be downloaded at: https://www.mdpi.com/article/10.3390/life12010099/s1, Figure S1. Establishment of a real-time fluorescence quantitative PCR detection method for target genes A: The CT diagram of the different reaction temperature for β-Actin, DDB2, AEN, TRIAP1 and TRAF4. B: The gradient amplification curve of plasmid standard for β-Actin, DDB2, AEN, TRIAP1 and TRAF4. The horizontal coordinate of CT is cycle numbers, the vertical coordinate of RFU is fluorescence intensity. Plasmid concentration of 1–8 is 6 × 108, 6 × 107, 6 × 106, 6 × 105, 6 × 104, 6 × 103, 6 × 102 and negative. C: Standard curves of 5 genes were plotted using the copy number de-log of plasmid standard as the x-coordinate and the Ct value as the y-coordinate. Supplementary Table S1. Differentially expressed genes in the three samples. Supplementary Table S2. Statistic of P53 signaling pathway related differentially expressed genes. Supplementary Table S3. RT-PCR primers and probes of human.
