# Supplementary figures and images for: Early Biomarkers Associated with P53 Signaling for Acute Radiation Injury
Source: Life (Basel). 2022 Jan 11;12(1):99. doi: 10.3390/life12010099 (PMC8778477; doi:10.3390/life12010099)

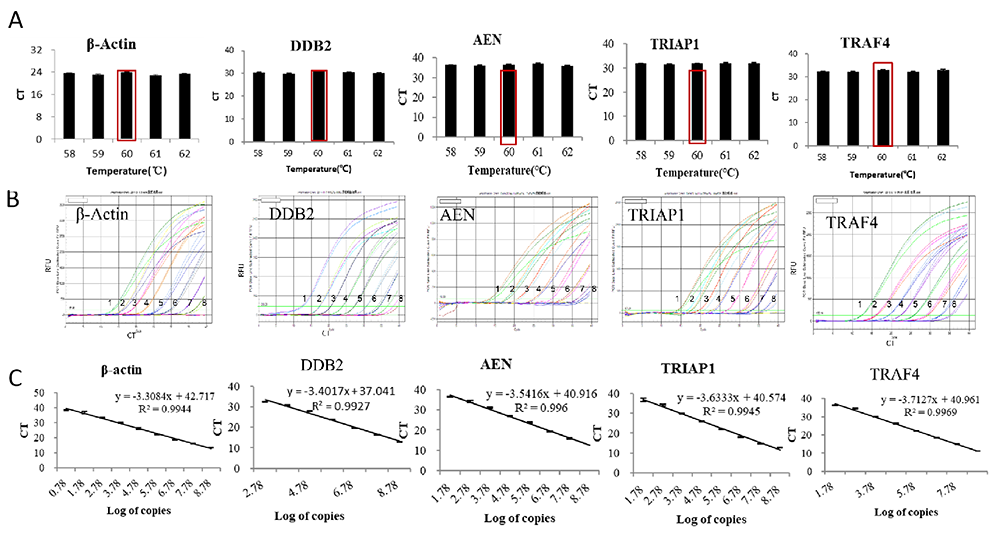

Supplement: Supplementary file 1 [file life-12-00099-s001.zip › supplementary figure S1.tif]
